# Secular Trends in Height, Body Mass, and BMI among Girls in the Eastern Poland Region (1986–2021): Public Health Perspectives

**DOI:** 10.2478/sjph-2026-0006

**Published:** 2026-03-01

**Authors:** Agnieszka Wasiluk, Jerzy Saczuk

**Affiliations:** Józef Piłsudski University of Physical Education in Warsaw, Faculty of Physical Education and Health, Biała Podlaska, Poland

**Keywords:** Secular trends, BMI, Physical development, Nutritional status, sekularni trendi, BMI, telesni razvoj, prehranski status

## Abstract

**Introduction:**

To assess long-term changes in body mass index (BMI) and weight status among girls from Eastern Poland between 1986 and 2021.

**Methods:**

Data were obtained from repeated cross-sectional, population-based surveys conducted in Eastern Poland in 1986, 1996, 2006, 2016, and 2021. The study included 14,825 girls aged 8, 13, and 17 years, recruited from the same schools across survey waves. Body height and body mass were measured by trained personnel using standardised procedures, and BMI was calculated. Weight status categories (underweight, normal weight, overweight and obesity) were defined using international BMI cut-off points. Statistical analyses included analysis of variance and post hoc comparisons.

**Results:**

Between 1986 and 2021, the largest increase in BMI was observed among 13-year-old girls (+1.66 kg/m^2^), followed by 8-year-olds (+1.14 kg/m^2^), while a decrease occurred among 17-year-olds (−1.13 kg/m^2^). The prevalence of underweight declined among 8- and 13-year-olds by 2.70 and 3.15 percentage points, respectively, but increased among 17-year-olds by 1.85 percentage points. In parallel, the combined prevalence of overweight and obesity increased across all age groups: 19.89 percentage points among 8-year-olds, 10.66 among 13-year-olds, and 3.87 among 17-year-olds, with the greatest increases occurring in recent survey periods.

**Conclusions:**

Over the past 35 years, BMI distribution among girls in Eastern Poland has shifted towards higher values, accompanied by a rise in overweight and obesity. The increase in underweight among older adolescents may reflect psychosocial pressures. These findings highlight the need for age-specific public health strategies addressing both excessive and insufficient body mass.

## Izvleček

**Uvod:**

Namen raziskave je bil oceniti dolgoročne spremembe indeksa telesne mase (ITM) in prehranskega statusa deklic iz vzhodne Poljske v obdobju 1986–2021.

**Metode:**

Antropometrični podatki so bili pridobljeni iz ponavljajočih se presečnih, populacijsko zasnovanih raziskav, izvedenih v vzhodni Poljski v letih 1986, 1996, 2006, 2016 in 2021. V raziskavo je bilo vključenih 14.825 deklic, starih 8, 13 in 17 let, ki so bile v vseh raziskovalnih obdobjih rekrutirane iz istih šol. Telesno višino in telesno maso so izmerili usposobljeni merilci po standardiziranih postopkih, ITM pa je bil izračunan na podlagi dobljenih vrednosti. Prehranski status (podhranjenost, normalna telesna masa, prekomerna telesna masa in debelost) je bil določen na podlagi mednarodnih mejnih vrednosti ITM. Statistična analiza je vključevala analizo variance (ANOVA) in post-hoc primerjave.

**Rezultati:**

Največje povečanje ITM v obdobju 1986–2021 je bilo ugotovljeno pri 13-letnih deklicah (+1,66 kg/m^2^), sledile so 8-letnice (+1,14 kg/m^2^), medtem ko je bil pri 17-letnicah zaznan upad ITM (−1,13 kg/m^2^). V celotnem opazovanem obdobju se je pogostost podhranjenosti zmanjšala pri 8-in 13-letnih deklicah za 2,70 oziroma 3,15 odstotne točke, medtem ko se je pri 17-letnicah povečala za 1,85 odstotne točke. Sočasno se je skupna pogostost prekomerne telesne mase in debelosti izrazito povečala v vseh starostnih skupinah, in sicer za 19,89 odstotne točke pri 8-letnicah, 10,66 odstotne točke pri 13-letnicah ter 3,87 odstotne točke pri 17-letnicah. Najizrazitejša povečanja so bila zabeležena v najnovejših raziskovalnih obdobjih.

**Zaključki:**

V zadnjih 35 letih se je porazdelitev ITM premaknila proti višjim vrednostim, skupaj z naraščanjem prekomerne telesne mase in debelosti. Povečanje podhranjenosti med starejšimi mladostnicami lahko odraža psihosocialne pritiske. Opažene spremembe imajo pomembne posledice za javno zdravje in poudarjajo potrebo po starosti prilagojenih preventivnih strategijah, ki obravnavajo tako prekomerno kot prenizko telesno maso.

## 1 INTRODUCTION

The analysis of long-term changes in basic anthropometric characteristics, such as body mass and stature, serves as a sensitive indicator of population health and overall living conditions (1). Throughout the 20th and 21st centuries, countries across the world, including Europe and Eurasia, have reported a significant increase in average height among children and adolescents, reflecting improvements in living standards (2, 3). Changes in body mass are more complex, as they result from the interaction between biological growth processes and lifestyle-related factors, such as dietary patterns, physical activity, and environmental conditions (including socio-economic circumstances and urbanisation), often leading to disturbances in weight-for-height proportions and an increased risk of underweight, overweight, and obesity. It should also be acknowledged that body height is influenced not only by environmental exposures but also by biological factors and nutritional status, particularly during critical growth periods. The so-called double burden of malnutrition now affects around 69–70% of countries worldwide, with obesity contributing most to the rise in abnormal body mass prevalence (4, 5). Global projections, including those from the Global Burden of Disease initiative, suggest that overweight and obesity among children and adolescents will continue to rise, potentially reaching critical levels by 2050 (5). Growth and body composition in children vary by age and developmental stage, but broader contextual factors, including geographic location and socio-economic conditions, also shape them. Therefore, region-specific monitoring of growth patterns and nutritional status is essential, particularly in populations exposed to uneven socio-economic development over time (6).

Within the broader Central and Eastern European context, Poland is particularly relevant due to its distinct historical and socio-economic background. Historically, it belonged to areas characterised by lower socio-economic development, largely due to its peripheral location along the former eastern border of the socialist bloc, limited industrialisation, and a predominantly rural settlement structure. Following the political and economic transformation after 1989, regional disparities persisted, as evidenced by slower economic growth, lower average incomes, and higher unemployment rates compared with the central and western parts of the country (7, 8). Although Poland’s accession to the European Union in 2004 initiated gradual improvements in infrastructure and living conditions, socio-economic inequalities between regions have remained evident. Local conditions are also important determinants of physical growth and nutritional status, as studies from Central and Eastern Europe (Slovenia, North Macedonia, the Czech Republic, Slovakia) show significant variations in the prevalence of overweight and obesity, as well as in body composition, depending on place of residence and social environment (9,10,11,12). Similar patterns are observed in high-income countries, such as Germany and Spain, where excessive body mass is strongly associated with environmental factors, lifestyle, and family socio-economic status (13, 14). The COVID-19 pandemic and associated restrictions on physical activity have exacerbated pre-existing disturbances in body weight and body mass index (BMI), thereby accelerating adverse public health trends among children and adolescents (15). In eastern Poland, the coexistence of underweight and excessive body mass reflects uneven socio-economic transformations (16). A regional study based on repeated cross-sectional surveys conducted between 1986 and 2016 demonstrated that the highest prevalence of both underweight and excessive body mass was observed among the youngest girls, which declined with increasing age (17). Given these observations and the dynamic changes in living conditions in Poland—particularly following accession to the European Union and during the COVID-19 pandemic— systematic monitoring of growth and nutritional status among girls from socio-economically disadvantaged backgrounds is crucial. In addition, nationwide family support policies, such as the “Family 500+” programme (18) providing financial benefits to households with children, may further modify living conditions and nutritional patterns, underscoring the need for long-term population-based monitoring. This study aimed to assess long-term changes in body height, body mass, and body mass index (BMI) among girls aged 8, 13, and 17 years living in eastern Poland between 1986 and 2021.

## 2 METHODS

The present study is part of a long-term research project initiated in 1986 in eastern Poland and based on repeated cross-sectional surveys. Earlier results from this project, including analyses of girls and boys from the same regions, as well as a detailed description of the measurement methodology, have been published previously (17, 19). The current analysis extends these findings by including data from the most recent survey conducted in 2021.

### 2.1 Study design and time frame

In 1986, a population-based study was conducted involving children and adolescents aged 7–18 years from the eastern regions of Poland as part of a national research programme aimed at monitoring biological development and health-related characteristics of the Polish child and adolescent population. The Eastern Poland region was defined as the historical eastern borderland of the country; all sub-regions constituting this area were included in the study, and no eligible sub-regions were excluded. The study had a repeated cross-sectional design. Subsequent survey waves were conducted in the same areas in 1996, 2006, 2016, and 2021 as part of statutory research programmes. Although the surveys included both boys and girls, the present analysis is based exclusively on data obtained from girls. All girls aged 8, 13, and 17 years with available anthropometric measurements from each survey year were included in the analyses to ensure consistent age-group comparisons across the entire observation period. In all survey waves, recruitment was conducted in schools within the study regions. Seventy schools were randomly selected for inclusion based on official lists obtained from the Regional Education Authorities, reflecting the region’s settlement structure. Eligible participants were girls residing in eastern Poland who attended the selected schools and whose parents or legal guardians provided written informed consent for participation. Children who did not meet the residency criterion or whose parents did not provide consent were not included. The present analyses are based on a subset of data collected within a broader research project. Information on participation rates was not systematically recorded across all survey waves. Although sample composition differed across survey years in terms of sample size and age distribution, the use of the same schools and identical inclusion criteria ensured environmental consistency and comparability over time. Differences in sample size across survey waves reflected demographic changes, including variations in birth cohort sizes and the school-age population, differences in school enrolment, and differences in the availability of complete anthropometric data for the predefined age groups. Sample sizes for each survey wave are reported in Table 1. For 2021, the dataset of 948 girls was supplemented with 1,861 participants recruited within the programme “Active Return to School – PE with AWF,” conducted in the Podlaskie, Lubelskie, and Podkarpackie voivodeships (first-level administrative regions of Poland). Data collection was carried out in the same schools as in previous survey waves. Girls aged 8, 13, and 17 years who met the same inclusion criteria as in earlier surveys were selected for anthropometric measurements. The different organisational framework did not affect the sampling procedure or measurement protocol.

**Table 1.** Number of girls examined by chronological age and observation period.

**Year**	**Age 8**	**Age 13**	**Age 17**	**Total**
**1986**	1776	2488	2553	6817
**1996**	159	269	101	529
**2006**	788	1209	1542	3539
**2016**	344	533	304	1181
**2021**	1566	763	430	2759
**Total**	4633	5262	4930	14825

### 2.2 Sampling procedure, data collection process, and inclusion criteria

For the present analysis, the study included 14,825 girls divided into three age groups reflecting stages of biological development: prepubertal (8 years, n = 4,633), pubertal (13 years, n = 5,262), and postpubertal (17 years, n = 4,930) (Table 1).

Age at the time of anthropometric measurement was calculated based on birth dates obtained from questionnaires and expressed as a decimal value, which was then categorised into full years (e.g., 8 years: 7.50–8.49; analogously for other age groups).

### 2.3 Observed outcome

Anthropometric measurements were performed by trained research staff according to standardised procedures recommended for field studies in human biology (20). These procedures included clearly defined body positioning and the use of standard anthropometric landmarks to ensure consistent placement of measurement instruments, a standardised measurement sequence, and instrument calibration. The anatomical landmarks were used solely to standardise measurement procedures and were not analysed as separate variables. All measurements were conducted at the participating schools under uniform conditions, ensuring methodological consistency and comparability of results across survey waves.

Body height was measured to the nearest 0.1 cm using a portable stadiometer, and body weight was measured to the nearest 0.1 kg using calibrated electronic scales, with participants wearing light clothing and no shoes. Body mass index (BMI, kg/m^2^) was calculated as body weight in kilograms divided by the square of body height in metres.

### 2.4 Methods of analysis

Means and variability of height, weight, and BMI were calculated for each age group and survey year. Secular changes over time were assessed by comparing measurements across five survey years (1986, 1996, 2006, 2016, and 2021). Overall differences between measurement years within each age group were examined using one-way analysis of variance (ANOVA). When the ANOVA indicated statistically significant differences, pairwise comparisons between study years were performed using a Bonferroni-adjusted post hoc test to control for Type I error. Statistical significance was set at p≤0.05.

Participants were classified into three nutritional status categories according to the international BMI cut-off points proposed by Cole (21) and Cole et al. (22): underweight (grades I–III combined), normal weight, overweight, and obesity (overweight and obesity combined). The prevalence (frequencies) of each nutritional status category was calculated separately for each age group and survey year. Differences in the distribution of nutritional status categories across survey years were assessed using the χ^2^ test, with statistical significance set at p ≤ 0.05.

### 2.5 Ethical considerations

The study was conducted in accordance with the principles of the Declaration of Helsinki. Ethical approval for the surveys conducted in 1986, 1996, and 2006 was obtained from the Faculty Ethics Committee within the framework of the State Key Problem 10.7 (1986) and the internal research projects D.S. 49 (1996) and D.S. 203 (2006), at the project approval stage. Research conducted in 2016 and 2021 was approved by the Ethics Committee of the Józef Piłsudski University of Physical Education in Warsaw (approval number: SKE 01-13/2014; approved on 30 June 2014). Written informed consent for participation in the study was obtained from the legal guardians of all underage participants, and participation of children and adolescents was voluntary.

## 3 RESULTS

### 3.1 Secular trends in height, body mass, and BMI

Between 1986 and 2021, an increase in mean height was observed across all three investigated age groups of girls from the eastern regions of Poland (Table 2). Most comparisons between consecutive survey periods showed statistically significant differences, indicating a clear but non-linear pattern of change over time. The magnitude of change was calculated as the difference between mean height values recorded in 2021 and 1986 for each age group. The greatest increase was observed among 13-year-old girls (+7.10 cm), followed by 8-year-olds (+6.13 cm) and 17-year-olds (+4.71 cm). In the group of 8-year-old girls, despite a slight initial decrease in mean height between 1986 and 1996 (−1.27 cm), a consistent increase was observed in subsequent periods. Between 1996 and 2006, mean height increased by +3.08 cm, between 2006 and 2016 by +1.66 cm, and between 2016 and 2021 by +2.66 cm, indicating a sustained upward trend over time. Among 13-year-old girls, mean height increased by +2.61 cm during the second decade of the study (1996–2006). This was followed by a pronounced decline of −1.95 cm between 2006 and 2016, and subsequently by the largest increase observed in the final period (2016–2021), amounting to +4.64 cm. In 17-year-old girls, height changes showed a less uniform pattern. Increases were observed between 1996 and 2006 (+3.08 cm) and between 2006 and 2016 (+1.41 cm); however, these changes did not follow a consistent decade-based trend.

**Table 2.** Height of girls examined between 1986 and 2021, including results of one-way ANOVA and Bonferroni-adjusted post hoc test.

**Age**	**Year**														

	**1986 (I)**			**1996 (II)**			**2006 (III)**			**2016 (IV)**			**2021 (V)**		

	**n**	**Mean**	**SD**	**n**	**Mean**	**SD**	**n**	**Mean**	**SD**	**n**	**Mean**	**SD**	**n**	**Mean**	**SD**
**8**	1776	127.75	5.93	159	126.48	7.41	788	129.56	6.86	344	131.22	6.53	1566	133.88	6.41
**13**	2488	154.53	6.89	269	156.33	7.32	1209	158.94	8.30	533	156.99	7.68	763	161.63	6.61
**17**	2553	162.14	5.91	101	163.46	4.77	1542	166.54	6.19	304	167.95	5.94	430	166.85	6.32

**values of one-way ANOVA and Bonferroni-adjusted post hoc test**											

	**ANOVA**	**I-II**	**I-III**	**I-IV**	**I-V**	**II-III**	**II-IV**	**II-V**	**III-IV**	**III-V**	**IV-V**
**8**	214.35	3.41	9.41^*^	13.10^*^	39.34^*^	39.34^*^	11.00^*^	19.78^*^	5.91^*^	22.00^*^	13 9.94^*^
**13**	169.56	5.43	24.36^*^	9.98^*^	3.23^*^	3.23^*^	1.71	14.48^*^	7.26^*^	11.27^*^	15.92^*^
**17**	183.35	3.06	32.07^*^	22.51^*^	21.24^*^	21.24^*^	9.19^*^	7.21^*^	5.28^*^	1.34	3.45

Legend: n – number of participants; mean – arithmetic mean; SD – standard deviation; I–V – study years: I = 1986, II = 1996, III = 2006, IV = 2016, V = 2021; I–II, I–III, … – comparisons between corresponding study years (e.g., I–II = 1986 vs. 1996). The values in the lower part of the table represent test statistics from Bonferroni-adjusted pairwise post hoc comparisons following a one-way ANOVA.

Statistically significant differences at p ≤ 0.05 are marked with an asterisk.

The greatest increase in body mass over the entire study period, calculated as the difference between mean values recorded in 2021 and 1986, was observed among 13-year-old girls (+8.71 kg). This was followed by the 8-year-old group (+4.93 kg), whereas the smallest overall change was found in 17-year-olds (+0.36 kg). Comparisons between survey years within each age group indicated that pronounced changes occurred primarily in the prepubertal and pubertal groups, while changes among 17-year-olds were smaller and less consistent (Table 3). In 8-year-old girls, body mass remained relatively stable between 1986 and 2006, with no significant differences between consecutive survey years. Clear increases were observed in later periods, amounting to +1.69 kg between 2006 and 2016 and +3.11 kg between 2016 and 2021. Among 13-year-olds, changes in body mass varied across survey periods. A small increase was noted between 1986 and 1996 (+0.29 kg), followed by a marked rise between 1996 and 2006 (+2.94 kg). This was followed by a modest decrease between 2006 and 2016 (−0.74 kg) and, subsequently, by a pronounced increase between 2016 and 2021 (+6.22 kg). In the oldest age group, body mass showed relatively minor fluctuations over time. A slight decrease was observed between 1986 and 2006, followed by an increase of +1.48 kg between 2006 and 2016. In the final observation period (2016–2021), body mass increased only slightly (+0.22 kg).

**Table 3.** Body mass of girls examined between 1986 and 2021, including results of one-way ANOVA and Bonferroni-adjusted post hoc test.

**Age**	**Year**														

	**1986 (I)**			**1996 (II)**			**2006 (III)**			**2016 (IV)**			**2021 (V)**		

	**n**	**Mean**	**SD**	**n**	**Mean**	**SD**	**n**	**Mean**	**SD**	**n**	**Mean**	**SD**	**n**	**Mean**	**SD**
**8**	1776	26.92	4.14	159	26.42	5.43	788	27.05	5.22	344	28.74	6.25	1566	31.85	7.72
**13**	2488	44.85	8.52	269	45.14	8.02	1209	48.08	8.23	533	47.34	8.60	763	53.56	10.33
**17**	2553	57.85	4.88	101	57.17	7.31	1542	56.51	7.24	304	57.99	6.92	430	58.21	9.01

**values of one-way ANOVA and Bonferroni-adjusted post hoc test**											

	**ANOVA**	**I-II**	**I-III**	**I-IV**	**I-V**	**II-III**	**II-IV**	**II-V**	**III-IV**	**III-V**	**IV-V**
**8**	171.11	1.41	0.72	7.36^*^	33.87^*^	1.73	5.76^*^	15.54^*^	6.23^*^	26.17^*^	12.44^*^
**13**	153.21	0.73	14.93^*^	8.46^*^	34.11^*^	7.07^*^	4.77^*^	19.25^*^	2.31	19.21^*^	17.86^*^
**17**	13.29	1.50	9.31^*^	0.52	1.55	1.44	1.60	2.11	5.28	6.98	0.66

Legend: n – number of participants; mean – arithmetic mean; SD – standard deviation; I–V – study years: I = 1986, II = 1996, III = 2006, IV = 2016, V = 2021; I–II, I–III, … – comparisons between corresponding study years (e.g., I–II = 1986 vs. 1996). The values in the lower part of the table represent test statistics from Bonferroni-adjusted pairwise post hoc comparisons following a one-way ANOVA.

Statistically significant differences at p ≤ 0.05 are marked with an asterisk.

Over the entire study period, the largest increase in body mass index (BMI), calculated as the difference between mean values recorded in 2021 and 1986, was observed in 13-year-old girls (+1.66 kg/m^2^). In the group of 8-year-olds, BMI increased by +1.14 kg/m^2^, whereas in the 17-year-olds, a decrease of −1.13 kg/m^2^ was observed (Table 4). Comparisons between consecutive survey periods within each age group revealed distinct temporal patterns. In 8-year-old girls, BMI values remained relatively stable during the first two observation periods (1986–1996: +0.02 kg/m^2^; 1996–2006: −0.41 kg/m^2^). Clear increases were observed in later periods, amounting to +0.50 kg/m^2^ between 2006 and 2016 and +1.03 kg/m^2^ between 2016 and 2021. Among 13-year-olds, BMI decreased between 1986 and 1996 (−0.31 kg/m^2^), followed by an increase of +0.56 kg/m^2^ between 1996 and 2006. This upward trend continued in subsequent survey periods, with an increase of +0.10 kg/m^2^ between 2006 and 2016 and a pronounced rise of +1.31 kg/m^2^ between 2016 and 2021. In the oldest age group, BMI changes varied over time. After decreases observed between 1986 and 1996 (−0.61 kg/m^2^) and between 1996 and 2006 (−1.03 kg/m^2^), BMI increased by +1.16 kg/m^2^ between 2006 and 2016, followed by a decrease of −0.65 kg/m^2^ in the final observation period (2016–2021).

**Table 4.** BMI of girls examined between 1986 and 2021, including results of one-way ANOVA and Bonferroni-adjusted post hoc test.

**Age**	**Year**														

	**1986 (I)**			**1996 (II)**			**2006 (III)**			**2016 (IV)**			**2021 (V)**		

	**n**	**Mean**	**SD**	**n**	**Mean**	**SD**	**n**	**Mean**	**SD**	**n**	**Mean**	**SD**	**n**	**Mean**	**SD**
**8**	1776	16.50	1.71	159	16.52	1.87	788	16.11	2.08	344	16.61	2.89	1566	17.64	3.41
**13**	2488	18.78	2.09	269	18.47	2.31	1209	19.03	2.33	533	19.13	2.71	763	20.44	3.42
**17**	2553	22.01	1.88	101	21.40	1.76	1542	20.37	1.74	304	21.53	1.92	430	20.88	2.84

**values of one-way ANOVA and Bonferroni-adjusted post hoc test**											

	**ANOVA**	**I-II**	**I-III**	**I-IV**	**I-V**	**II-III**	**II-IV**	**II-V**	**III-IV**	**III-V**	**IV-V**
**8**	63.63	0.13	5.04^*^	1.03	18.20^*^	2.61	0.52	7.45^*^	4.28	19.39^*^	9.57^*^
**13**	72.12	2.78	4.11^*^	4.23^*^	23.11^*^	4.79^*^	5.08^*^	16.01^*^	1.11	17.57^*^	13.37^*^
**17**	178.88	4.38^*^	37.03^*^	5.76^*^	15.79^*^	7.30^*^	0.82	3.42	13.46^*^	6.81^*^	6.32^*^

Legend: n – number of participants; mean – arithmetic mean; SD – standard deviation; I–V – study years: I = 1986, II = 1996, III = 2006, IV = 2016, V = 2021; I–II, I–III, … – comparisons between corresponding study years (e.g., I–II = 1986 vs. 1996). The values in the lower part of the table represent test statistics from Bonferroni-adjusted pairwise post hoc comparisons following a one-way ANOVA.

Statistically significant differences at p ≤ 0.05 are marked with an asterisk.

### 3.2 Prevalence of underweight, normal weight, overweight and obesity

**Figure 1.**
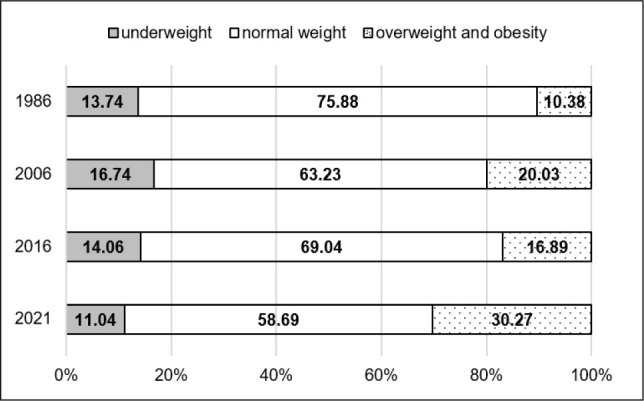
Prevalence (%) of underweight, normal weight, overweight and obesity among 8-year-old girls in 1986, 2006, 2016, and 2021.

**Figure 2.**
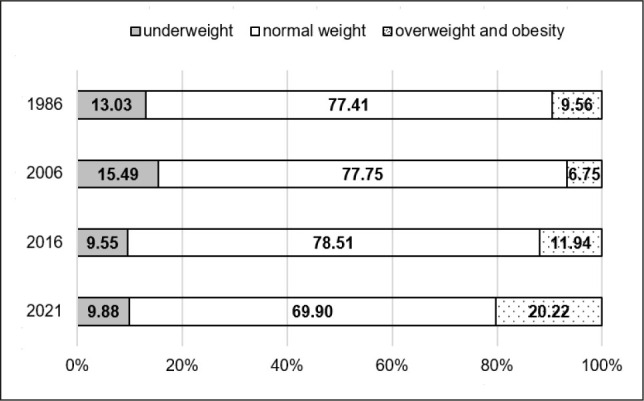
Prevalence (%) of underweight, normal weight, overweight and obesity among 13-year-old girls in 1986, 2006, 2016, and 2021.

**Figure 3.**
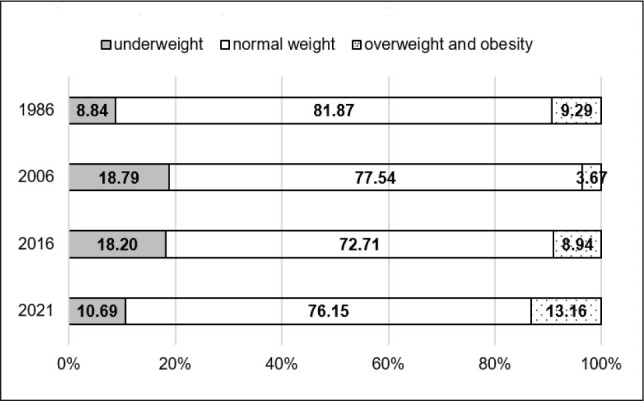
Prevalence (%) of underweight, normal weight, overweight and obesity among 17-year-old girls in 1986, 2006, 2016, and 2021.

The secular trends described above, based on mean BMI values, do not fully capture the variability in nutritional status among the girls studied, as mean values may mask changes at the extremes of the BMI distribution, such as shifts in the prevalence of underweight, overweight, and obesity. Therefore, additional analyses were conducted to examine changes in the distribution of nutritional status categories. The results of these analyses are presented in Table 5 and Figures 1–3. The prevalence of underweight, normal weight, overweight and obesity was analysed separately for each age group (8, 13, and 17 years) and survey year. Due to the unavailability of individual-level data for 1996, including the absence of individual BMI values, weight-status classification (underweight, normal weight, overweight, and obesity) could not be performed for this survey year; therefore, 1996 was excluded from these analyses. Between 1986 and 2021, substantial changes in the distribution of BMI categories were observed across all age groups. In girls aged 8 and 13 years, the prevalence of underweight decreased by −2.70% and −3.15%, respectively, whereas in 17-year-old girls a slight increase of +1.85% was noted over the same period. The largest reductions in underweight prevalence were observed among 8-year-olds between 2006 and 2021 (−5.70%) and among 13-year-olds between 2006 and 2016 (−5.94%). In the oldest age group, underweight prevalence increased between 1986 and 2006 (+8.84%), followed by a decrease in the final observation period (2016–2021; −7.51%). Across all age groups, a consistent decline in the proportion of girls classified as having normal body weight was observed. Between 1986 and 2021, this proportion decreased by −17.19% among 8-year-olds, −7.52% among 13-year-olds, and −5.72% among 17-year-olds. Statistically significant changes were observed primarily in the youngest age group, particularly between 1986 and 2006 (−12.65%). The most pronounced and consistent trend was an increase in the prevalence of overweight and obesity in all age groups. Over the entire study period, this increase amounted to +19.89% among 8-year-olds, +10.66% among 13-year-olds, and +3.87% among 17-year-olds. The largest increases were observed among 8-year-olds between 2016 and 2021 (+13.38%) and among 13- and 17-year-olds between 2006 and 2021 (+13.47% and +9.49%, respectively).

**Table 4.** Statistical significance of differences in the prevalence of underweight, normal weight, overweight and obesity across successive survey years within each age group.

	**1986–2006**	**1986–2016**	**1986–2021**	**2006–2016**	**2006–2021**	**2016–2021**
	**Age 8**					

**Underweight**	2.849	0.019	4.355^*^	0.956	11.150^*^	1.885
**Normal weight**	7.303^*^	1.063	21.864^*^	0.742	1.108	2.965
**Overweight and obesity**	31.067^*^	8.510^*^	143.567^*^	1.062	17.169^*^	16.439^*^

	**Age 13**					

**Underweight**	3.055	4.110^*^	4.432^*^	9.063^*^	10.194^*^	0.033
**Normal weight**	0.007	0.039	2.545	0.015	2.239	1.812
**Overweight and obesity**	7.135^*^	2.151	43.016^*^	10.275^*^	61.009^*^	11.482^*^

	**Age 17**					

**Underweight**	64.341^*^	17.791^*^	1.212	0.041	12.511^*^	6.256^*^
**Normal weight**	1.252	1.630	0.841	0.447	0.048	0.163
**Overweight and obesity**	44.020^*^	0.035	4.638^*^	12.172^*^	40.514^*^	2.573

Legend:

* Significant differences at the level of p ≤ 0.05.

## 4 DISCUSSION

This study demonstrated secular changes in height, body mass, and body mass index (BMI) among girls aged 8, 13, and 17 years from eastern Poland between 1986 and 2021, with patterns varying across survey periods and age groups. Overall, mean height increased in all age groups, accompanied by marked increases in body mass and BMI, particularly among younger girls. At the same time, the distribution of nutritional status shifted towards a higher prevalence of overweight and obesity, while underweight declined in younger age groups but increased among older adolescents.

In the present study, secular trends refer specifically to long-term changes in height, body mass, and body mass index (BMI) observed among Polish girls across successive survey waves. These secular trends are most commonly manifested as increases in average height and body mass, often accompanied by accelerated sexual maturation. Since the mid-20th century, increases in average height have been largely attributed to improvements in living conditions, including better nutrition, healthcare, and educational access (23, 24). The findings of the present study confirm the persistence of such height-related secular changes among girls from eastern Poland over a 35-year period. In contrast, the concurrent increases in body mass, BMI, and the prevalence of overweight and obesity likely reflect qualitative changes in dietary patterns and lifestyle behaviours rather than improved nutrition per se. The magnitude and direction of secular changes in height, body mass, and BMI are closely linked to socio-economic and environmental contexts, as demonstrated by both national (25) and international studies (26). In regions with lower levels of economic development, secular changes in growth tend to occur at a slower pace, whereas improvements in living standards are associated with a more rapid pace of growth-related changes. These terms refer to differences in the rate of change over time rather than to negative or positive evaluations of growth. In contrast, in high-income countries, increases in body mass that are not accompanied by proportional gains in height have been interpreted as a shift in secular trends towards overweight and obesity (27, 28).

In eastern Poland, long-term changes in physical growth did not follow a strictly linear pattern. Periods of marked height increases during the late 20th century were followed by pronounced increases in body mass and BMI in the early 21st century. This temporal pattern may be linked to broader socio-economic transformations in Poland, particularly the post-communist transition characterised by improved living conditions, increased food availability, and substantial changes in dietary patterns and lifestyle. Epidemiological evidence indicates a shift toward higher energy intake, greater consumption of processed foods, and reduced physical activity after the political and economic transition, which coincided with a rising prevalence of overweight and obesity in the Polish population (29, 30). While this association suggests a plausible link between socio-economic change and observed anthropometric trends, it should be interpreted as a hypothesis supported by population-level data rather than a direct causal relationship. In the most recent decade, these trends appeared to slow, which may suggest that genetic growth potential has largely been reached and that new environmental constraints have emerged, as proposed in earlier research (25). Each measurement point reflected distinct socio-economic conditions in Poland: 1986 corresponded to the final phase of the centrally planned economy; 1996 to the period of political and economic transition; 2006 to Poland’s accession to the European Union; 2016 to a phase of relative economic stabilisation; and 2021 to the COVID-19 pandemic. Although causal relationships cannot be established within the present study, these contextual shifts provide a plausible framework for interpreting temporal variation in growth patterns. Periods of economic instability may have contributed to greater variability in physical development, whereas subsequent stabilisation and improved access to food, healthcare, and education likely supported increases in body height, particularly among younger girls. At the same time, lifestyle changes, reduced physical activity, and increased availability of energy-dense foods may have promoted excessive body mass gain, especially in later survey periods, with these effects potentially amplified during the COVID-19 pandemic. An exception to the overall upward secular trend was the temporary decline in mean height observed among 8-year-old girls between 1986 and 1996, as well as the decrease noted among 17-year-old girls between 2016 and 2021. These findings should be interpreted with caution and are unlikely to indicate a true reversal of long-term growth trends at the population level. Short-term socio-economic instability, such as that observed during the early 1990s, would not be expected to substantially reduce mean height unless associated with widespread and prolonged health impairments. Alternative explanations for the observed decline among younger girls include measurement error, increased variability within the sample, or reduced sample sizes in specific survey waves, which may have influenced the estimates. The more recent decrease observed among older adolescents should be interpreted with caution and is unlikely to reflect a true reduction in attained height. Factors associated with the COVID-19 pandemic, such as disruptions to data collection, changes in sample composition, or increased variability, may have influenced the estimates. While pandemic-related lifestyle changes may have affected body mass and BMI, their impact on attained height in this age group is likely limited. Given the study’s repeated cross-sectional design, these interpretations should be regarded as hypotheses requiring further investigation.

In the context of the global obesity epidemic, increasing attention has been paid to body mass-to-height relationships rather than height alone. Monitoring BMI during puberty is particularly important, as longitudinal studies have shown that elevated BMI in adolescence constitutes an independent risk factor for cardiovascular morbidity in adulthood (31). In this regard, Bygdell et al. (31) demonstrated increasing variability in BMI among girls over several decades, highlighting the importance of analysing entire BMI distributions rather than mean values alone. Consistent with these observations, the present study revealed substantial shifts in BMI distribution among girls from eastern Poland. A decline in underweight prevalence among younger girls was accompanied by a reduction in the proportion with normal body weight and a pronounced increase in overweight and obesity, particularly in the youngest age group. Similar patterns have been reported in population-based studies from Germany, South Korea, and the United States (32,33,34). At the same time, recent European evidence suggests that long-term BMI trends may reflect a complex coexistence of underweight and overweight rather than a uniform shift towards obesity. Studies from Denmark (35) and Norway (36) have demonstrated that overweight, obesity, and thinness may coexist within the same populations and vary according to age, sex, region, and population density, underscoring the context-dependent nature of BMI-related trends. Although some economically developed countries have reported partial stabilisation of overweight and obesity prevalence among children and adolescents (32, 33), excessive body mass remains a major public health challenge. In eastern Poland, the increase in overweight and obesity observed in the most recent survey years may be related to pandemic-related restrictions, lifestyle changes, and broader socio-economic conditions. While social support programmes such as “Family 500+” (18) were introduced to improve household economic security, their potential impact on children’s nutritional status cannot be directly assessed within the present analysis.

Between 1986 and 2021, underweight prevalence decreased among girls aged 8 and 13 years, whereas an opposite trend was observed among 17-year-olds. While the reduction in underweight among younger girls could be interpreted as a partial improvement in nutritional status, global analyses indicate that population-level BMI shifts are driven primarily by increases in overweight and obesity rather than genuine improvements in nutritional adequacy (34). The increase in underweight among older adolescent girls may be associated with psychosocial pressures, unhealthy eating behaviours, and an elevated risk of eating disorders (37, 38), potentially influenced by biological maturation and environmental factors (39). However, given the design of the present study, these interpretations remain speculative.

The main strengths of this study include its long observation period spanning 35 years, the large sample size, and the focus on eastern Poland—a region underrepresented in population-based growth research. Nevertheless, several limitations should be acknowledged. The analysis was restricted to girls, limiting generalisability to the overall paediatric population. The repeated cross-sectional design precludes assessment of individual growth trajectories, and unequal sample sizes across survey years may have influenced variability. Additionally, the lack of individual-level data on diet, physical activity, and socio-economic status limits causal inference. Despite these limitations, the consistent application of standardised anthropometric procedures across all survey waves strengthens the validity and comparability of the findings. Temporal changes should nevertheless be interpreted cautiously, particularly in light of potential external influences such as the COVID-19 pandemic.

## 5 CONCLUSIONS

The results of this study demonstrate clear long-term secular trends in physical growth and nutritional status among girls from eastern Poland between 1986 and 2021. While mean body height continued to improve across successive cohorts, the most pronounced changes were observed in body mass and BMI. Specifically, there was a sustained shift in BMI distribution towards higher values, characterised by a decreasing prevalence of underweight among younger girls alongside a marked increase in the prevalence of overweight and obesity, particularly in the most recent survey years. In contrast, the increase in underweight prevalence among 17-year-old girls suggests that age-specific factors may influence nutritional status during late adolescence. The differing patterns observed indicate that changes in body weight status vary across developmental stages and do not follow a uniform trajectory. These findings have important public health implications and highlight the need for continued population-level monitoring. They also support the early implementation of age-appropriate health education and targeted preventive interventions addressing both excessive body mass and the risk of underweight and eating disorders among adolescent girls.
